# Emerging role of epigenetic mechanisms in glaucoma and their translational potential

**DOI:** 10.3389/fgene.2026.1781271

**Published:** 2026-02-23

**Authors:** Altaf A. Kondkar, Tahira Sultan, Taif A. Azad, Saleh A. Al-Obeidan

**Affiliations:** 1 King Saud University-Research Center for Excellence in Ophthalmology and Visual Sciences, Department of Ophthalmology, College of Medicine, King Saud University, Riyadh, Saudi Arabia; 2 Glaucoma Research Chair in Ophthalmology, College of Medicine, King Saud University, Riyadh, Saudi Arabia; 3 King Saud University Medical City, King Saud University, Riyadh, Saudi Arabia

**Keywords:** biomarkers, DNA methylation, epigenetics, glaucoma, histone modifications, neurodegeneration, non-coding RNA

## Abstract

Glaucoma, a leading cause of irreversible blindness, is a complex polygenic disease where significant clinical and genetic heterogeneity do not explain all glaucoma cases, highlighting the need for a deeper understanding of molecular mechanisms like epigenetics. This review examines the emerging role of key epigenetic mechanisms, specifically DNA methylation, histone modifications, and non-coding RNAs in glaucoma pathogenesis and their potential as biomarkers and therapeutic targets. We discuss how aberrant DNA methylation (e.g., *GDF7* hypomethylation/*CDKN2B* hypermethylation) promotes trabecular meshwork fibrosis and increases optic nerve vulnerability, contributing to disease development and/or progression. The *METTL23* histone methylation linked to retinal ganglion cell death at normal eye pressure, and disease-specific microRNA profiles further support the role of epigenetic involvement in glaucoma. The proof-of-concept studies of GDF7 neutralization in primate models and the OSK-factor reprogramming in aged and glaucoma mice models, show that epigenetic changes are reversible and can restore visual functions. DNA methylation-based epigenetic clocks identify glaucoma as an accelerated molecular aging process. Although promising, the current evidences are largely preclinical and long-term human data are still lacking. Nonetheless, the inherent reversible nature of epigenetics offers significant translational potential. Methylation, epigenetic clocks, and circulating microRNA profiles could enable early, non-invasive biomarkers for diagnosis and prognosis. Future efforts are needed to validate biomarkers in large cohorts and develop targeted epigenetic therapies. In conclusion, epigenetics is redefining our current understanding of glaucoma from a pressure-based disease to a modifiable link between genes and environment paving the way for personalized care for vision preservation beyond pressure-lowering treatments.

## 1 Introduction

Glaucoma is a group of optic neuropathies characterized by elevated intraocular pressure (IOP), progressive loss of retinal ganglion cells (RGCs), damage to the optic nerve, and blindness (Dietze et al., 2025; Weinreb et al., 2014). The estimated worldwide prevalence of adult-onset glaucoma is 3.5% in the population aged 40 years and above, affecting around 80 million people globally, with an estimated projection to nearly 112 million by 2040 (Allison et al., 2020; Tham et al., 2014; Al-Manjoumi et al., 2023). Since elevated IOP is considered a major risk factor for RGC death in glaucoma, the current clinical management is also focused on lowering IOP (Park et al., 2023). However, up to 40% or more of glaucoma patients do not exhibit high IOP but still suffer from progressive optic nerve damage, as seen in patients with normal-tension glaucoma (NTG) (Kim and Park, 2016). These cases demonstrate that there are several other non-IOP factors contributing to glaucoma development.

**FIGURE 1:**
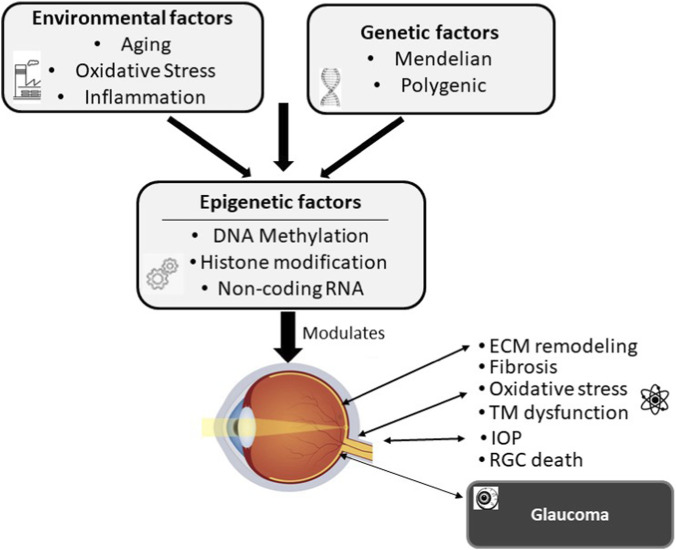
Conceptual model of epigenetics as the link between genetics and environment in the complex pathogenesis of glaucoma. Environmental factors interact with the genetic blueprint, and epigenetic mechanisms process these signals to regulate and influence crucial pathological outcomes, including extracellular matrix (ECM) remodeling, fibrosis, oxidative stress, trabecular meshwork (TM) dysfunction, intraocular pressure (IOP) alteration, leading to retinal ganglion cell (RGC) death and the eventual clinical manifestation of glaucoma.

Glaucoma is a complex multifactorial disease involving both genetic and environmental influences (Wiggs, 2012). A number of genetic studies, including genome-wide, have identified several Mendelian genes (e.g., *MYOC*, *CYP1B1*, *PAX6*, *FOXC1*), chromosomal loci (GLC1A-P), and DNA variants associated with glaucoma (Kolovos et al., 2025; Tirendi et al., 2023; Zukerman et al., 2020; Abu-Amero et al., 2015). Although these studies have provided significant mechanistic insights into disease pathogenesis, they fail to explain the clinical variability seen in glaucoma onset, progression, and treatment response, suggesting the role of factors beyond genetic determinants (D’Esposito et al., 2024; Wang and Wang, 2023). There is evidence to believe that the environmental factors, such as aging, oxidative stress, and inflammation, interact with the genome in complex ways, inducing aberrant epigenetic regulation that might be the missing link, beyond the genetic determinants (Figure 1) (Wang and Wang, 2023; Dolinoy and Jirtle, 2008; Medeiros et al., 2025).

Epigenetics refers to the heritable changes observed in gene expression without alteration in the DNA sequence (Kachhawaha et al., 2023). There is strong evidence to suggest that epigenetics might be associated with glaucoma development and progression, plausibly by altering gene expression profiles critical to IOP control, optic nerve integrity, and neuroinflammation. These biological processes are central to aqueous humor dynamics, RGC survival, immune responses, and extracellular matrix (ECM) remodeling, which are strongly implicated in glaucoma (Figure 1) (D’Esposito et al., 2024; Tonti et al., 2024).

Eye represents an excellent model for epigenetic research (Alkozi et al., 2017; Lanza et al., 2019). The purpose of this review is to examine the epigenetic contributions of DNA methylation, histone modifications, *N*6-methyladenosine (m6A) methylation, and non-coding RNAs in glaucoma pathogenesis and their clinical translational potential.

## 2 Epigenetic mechanisms in glaucoma

### 2.1 DNA methylation

DNA methylation is often referred to as ‘the epigenetic switch.’ It involves the addition of a methyl group to a cytosine base, prominently at CpG sites near gene promoters, causing gene silencing when hypermethylated or gene transcription when hypomethylated (Jin and Liu, 2018; Lim and Maher, 2010). Methylation is a tightly regulated process involving specific enzymes that are critical for developmental processes, cellular differentiation, and maintenance of genomic stability. These include the ‘writers’ and ‘maintainers,’ which are the DNA Methyltransferases (DNMTs), like DNMT1 for maintenance and DNMT3A/B for new establishment. The “erasers” are the Ten-Eleven Translocation (TET) enzymes, which control the on/off switch by oxidizing the methyl group (Dan and Chen, 2022). DNA methylation offers a dynamic interface between environmental stimuli (e.g., oxidative stress) and gene expression in ocular tissues. Aberrant expression of these enzymes can lead to abnormal methylation patterns and dysregulation of genes involved in key processes implicated in glaucoma pathogenesis, such as ECM remodeling, oxidative stress, and neurodegeneration (Bou Ghanem et al., 2024; Fernández-Albarral et al., 2024).

#### 2.1.1 Evidence of differential DNA methylation in glaucoma

Several studies have demonstrated aberrant DNA methylation in different types of glaucoma. Junemann et al. reported a significantly higher global DNA methylation in blood cells of POAG patients compared to controls and PXG patients (Junemann et al., 2014). The trabeculectomy sections from glaucomatous eyes were reported to have significant DNA hypomethylation of Alu repetitive elements in POAG and PACG, and significant DNA hypermethylation of *HERV-K* in POAG patients (Chansangpetch et al., 2018). These methylation changes were proposed to predispose the trabecular meshwork (TM) cells to a profibrotic phenotype (Chansangpetch et al., 2018).

In PXG, the *LOXL1* promoter is reported to be hypermethylated in lens capsule and human Tenon fibroblasts that correlated with reduced *LOXL1* expression and increased ECM cross-linking (Greene et al., 2020; Ye et al., 2015). Interestingly, treatment of human Tenon Fibroblasts with DNMT inhibitor, 5-aza-dC, restored LOXL1 expression in PXG fibroblasts (Greene et al., 2020). Conversely, Kapuganti et al. reported hypomethylation of the clusterin promoter in patients with PXG (Kapuganti et al., 2023). In another study, *CDKN2B* gene promoter hypermethylation at the 9p21 locus has been linked to NTG susceptibility in women (Burdon et al., 2018). *CDKN2B* silencing due to hypermethylaiton causes its suppression that may increase the optic nerve’s vulnerability to non-IOP stressors (Burdon et al., 2018). This study elegantly demonstrated how epigenetic errors through aberrant methylation can impact critical RGC survival pathways, leading to neurodegeneration even at normal IOP.

#### 2.1.2 Mechanistic evidence of DNA methylation in glaucoma

High IOP is the result of decreased outflow capacity at the level of the TM and Schlemm’s canal, the primary exit route of aqueous humor (Carreon et al., 2017). Dexamethasone (DEX)-treated primary human TM cells exhibited hypomethylation of the thrombospondin-1 (*THBS1*) promoter region and reduced transcript levels of 2 DNA methyltransferases (DNMTs), DNMT1 and DNMT3A (Choy et al., 2025). This change was consistent with DNA methylation inhibitors, 5-azacytosine (5-AC) or 5-aza-2′-deoxycytidine (5-aza-dC), inducing an increase in THBS1 protein levels, leading to the reduced outflow facility *ex vivo* and increased IOP *in vivo* adult male C57BL/6 J mice (Choy et al., 2025). These results highlight a clear mechanism in which DNMT downregulation and promoter hypomethylation alter THBS1 expression and influence ECM composition and aqueous outflow resistance. In glaucoma, fibrosis is one of the key mechanisms in glaucoma development and progression, as a result of ECM deposition in the TM at the anterior of the eye, and at the optic nerve head in the lamina cribrosa, leading to aqueous outflow resistance, elevated IOP, and subsequently glaucoma (Keller and Peters, 2022). The *GDF7* gene promoter region is aberrantly hypomethylated in glaucomatous TM samples and TET-dependent (Wan et al., 2021). Hypomethylation causes gene activation, which releases the brake, causing GDF7 protein overexpression, increasing proteins like α-SMA, promoting fibrosis which clogs the drainage pathway, raising IOP. This landmark study is a prime example of a targetable epigenetic mechanism in POAG. Crucially, in a therapeutic proof-of-concept, neutralizing GDF7 with antibodies reduced this fibrosis and improved outflow in a primate model. This study showed that GDF7 is a direct, reversible, and therapeutic targetable epigenetic cause of high IOP and fibrosis in POAG, offering a potential for a novel anti-fibrotic therapy (Wan et al., 2021). Likewise, glaucomatous Schlemm’s canal endothelial cells have been shown to exhibit distinct methylation profiles in genes (*TGFBR3*, *TBX3*, *TNXB1, DAXX*, and *PITX2*) enriched in pathways regulating outflow resistance. However, further studies are needed to validate these findings (Cai et al., 2020).

Taken together, these studies suggest a complex and gene-specific methylation landscape in glaucoma subtypes and illustrate how DNA methylation can act as a molecular switch modulating distinct cellular pathways, promoting ECM remodeling in PXG (via *LOXL1* or *CLU*) and influencing neuroprotective cell-cycle regulation in NTG (via *CDKN2B*), thereby contributing directly to disease susceptibility. It can thus be speculated that promoter methylation changes may represent potential blood-based biomarkers for glaucoma risk assessment and gender-specific susceptibility, as well as a valuable target for new treatments to mitigate glaucoma or fibrosis-related outflow resistance to restore normal TM function and outflow facility.

### 2.2 Epigenetic reprogramming

*In vivo* epigenetic reprogramming using Yamanaka OSK factors (Oct4, Sox2, Klf4) has shown promise in preclinical models. Reprogramming aged or glaucomatous RGCs reverses DNA methylation patterns to a youthful state, restoring a healthy transcriptome and visual function in mice. Transient OSK expression in glaucomatous eyes reversed vision loss in mice. The effect was dependent on DNA demethylases TET1 and TET2 (Lu et al., 2020). Moving beyond the initial proof-of-concept, a follow-up study provided compelling evidence of long-term safety and efficacy for vision recovery in glaucoma (Karg et al., 2023). The studies provide groundbreaking evidence of epigenetic reprogramming as a viable and sustainable approach for recovering lost vision in glaucoma with direct neural rescue and repair, moving us closer to a future where we can complement IOP-lowering.

### 2.3 DNA methylation-based EpiScores

Recent evidence suggests that DNA methylation (DNAm)-based EpiScores and GrimAge acceleration are positively associated with glaucoma risk, indicating that the disease may represent an accelerated molecular aging process (Medeiros et al., 2025; Jiang et al., 2025). EpiScore and GrimAge are epigenetic clock that predicts biological age and mortality risk based on DNAm patterns. Accelerated epigenetic aging compared to chronological aging is associated with a 15% higher odds of faster glaucoma progression. Importantly, this relationship remains strong even in patients with relatively low IOP, indicating that epigenetic aging may predispose the optic nerve to damage from things like oxidative stress, independent of IOP level (Medeiros et al., 2025). These results highlight the potential clinical importance of epigenetic age acceleration as a non-invasive predictor of progression.

### 2.4 Histone modifications

Histone modifications, including acetylation, methylation, phosphorylation, and ubiquitination, can regulate gene expression by altering chromatin structure (Davie and Chadee, 1998; Zhang et al., 2015). These modifications, catalyzed by specific enzymes, play significant roles in regulating genes involved in maintenance of IOP and RGC health, thereby influencing glaucoma risk and severity (Tonti et al., 2024; Feng et al., 2023).

Studies on glaucoma-related histone modifications have mainly focused on deacetylation (Pelzel et al., 2010; Schmitt et al., 2014; Alsarraf et al., 2014; Sharma et al., 2016). Histone deacetylation functions as a crucial epigenetic switch that may accelerate glaucomatous neurodegeneration (Pelzel et al., 2010; Sharma et al., 2016; Biermann et al., 2011). The HDAC inhibitors have consistently demonstrated to exhibit neuroprotective effects and improved surgical outcomes in different animal models of glaucoma, highlighting their potential utility as pharmacological agents in glaucoma (Feng et al., 2023; Schmitt et al., 2014; Alsarraf et al., 2014; Sharma et al., 2016; Biermann et al., 2011).

Histone methylation is another critical regulatory mechanism in glaucoma. The trimethylation mark H3K27me3, catalyzed by EZH2 are both detected in RGCs (Rao et al., 2012). A study by Rao et al. (2010) showed that inhibition of EZH2 induces RGC apoptosis, underscoring the essential role of EZH2-mediated H3K27me3 in retinal neuronal survival and suggesting its involvement in glaucomatous degeneration (Rao et al., 2010).

#### 2.4.1 METTL23 and NTG: a histone methylation connection

Mutations in *the METTL23* gene encoding a histone arginine methyltransferase have been linked to NTG (Pan et al., 2022). METTL23 catalyzes the dimethylation of H3R17 in the retina. The c.A23G mutation inherited as an autosomal dominant condition results in METTL23 loss of function, leading to impaired dimethylation of histone H3R17. This disrupts gene regulation in RGCs, causing RGC apoptosis through NF-κB signaling. The study highlights a novel epigenetic etiology in NTG and provides a direct evidence linking abnormal histone methylation to glaucomatous neurodegeneration (Pan et al., 2022; Scheetz et al., 2024; Liu and Sun, 2022). In addition, the findings open new therapeutic avenues to target the downstream inflammatory NF-κB signaling or the histone methylation modulators, shifting the paradigm beyond the conventional IOP-based approach of glaucoma management.

### 2.5 N6-methyladenosine (m6A) modification

m6A is a common mRNA modification regulating RNA stability, splicing, translation, and decay, affecting gene expression (Jiang et al., 2021; Patil et al., 2018). The modification involves regulation by writers (e.g., methyltransferase-like protein 3, METTL3), erasers (e.g., fat mass and obesity-associated, FTO), and readers (e.g., YTH family protein YTHDC2) (Jiang et al., 2021). These writer, eraser, and reader proteins play a significant role in various diseases, but are less studied in glaucoma.

In PXG, elevated global m6A levels and upregulated writer and reader enzymes (e.g., METTL3, YTHDC2) in aqueous humor may serve as biomarkers, with m6A-modified transcripts enriched in matrix organization pathways (D’Esposito et al., 2024; Guan et al., 2023). In another study, differential m6A-methylated lncRNAs in PXG aqueous humor influenced glaucoma-related genes and processes (Guan et al., 2024). In POAG, bioinformatics analyses identified differentially expressed m6A regulators (e.g., upregulated reader YTHDF1, downregulated reader YTHDC2) in TM tissues. Silencing YTHDC2 in human TM cells enhanced migration and ECM synthesis, demonstrating a functional role in outflow resistance (Zhang et al., 2025). Current studies suggest that targeting writer and reader pathways could lead to new treatments. For example, blocking the writer METTL3 might control fibrosis in PXG, and silencing readers like YTHDC2 may prevent TM dysfunction in POAG (Tonti et al., 2024; Guan et al., 2024; Zhang et al., 2025).

To enhance clinical utility and highlight the diagnostic potential of the epigenetic changes discussed in the above sections, a summary of the key markers characterized by their subtype-specific roles in POAG, PXG, and NTG is listed in Table 1.

**TABLE 1:** Summary of key subtype-specific epigenetic markers in glaucoma.

Glaucoma subtype	Epigenetic mechanism	Specific marker	Change	References
PXG	DNA Methylation	*LOXL1* Promoter	Hypermethylation	Greene et al. (2020), Ye et al. (2015)
POAG	DNA Methylation	*GDF7* Promoter	Hypomethylation	Wan et al. (2021)
NTG	DNA Methylation	*CDKN2B* Promoter	Hypermethylation	Burdon et al. (2018)
NTG	Histone Modification	*METTL23*	Loss of Function	Pan et al. (2022)
PXG	m6A Methylation	*METTL3* (Writer)	Upregulated	Guan et al. (2023)
POAG	m6A Methylation	*YTHDC2* (Reader)	Downregulated	Zhang et al. (2025)

Abbreviations: PXG, pseudoexfoliation glaucoma; POAG, primary open-angle glaucoma; NTG, normal-tension glaucoma.

### 2.6 Noncoding RNAs

Noncoding RNAs (ncRNAs) like microRNAs (miRNAs), long non-coding RNAs (lncRNAs), and circular RNAs (circRNAs) are pivotal players in cell regulatory networks across a broad spectrum of biological processes implicated in glaucoma (Rong et al., 2021; Costa et al., 2025). Differential expression and abnormal function of these regulatory molecules can affect gene networks related to fibrosis, IOP, and optic nerve health, making them promising biomarker candidates and drug targets (Rong et al., 2021; Zhang et al., 2021; Huang et al., 2022).

#### 2.6.1 MiRNAs

miRNAs are the most extensively studied class of ncRNAs in glaucoma and have been elegantly reviewed elsewhere for detailed molecular summaries (Dobrzycka et al., 2023; Greene et al., 2022; Martinez and Peplow, 2022). Several studies have confirmed that these small RNA molecules (∼19–23 nucleotides in length) regulate a wide range of pathological processes, including ECM remodeling, TM cell function, RGC apoptosis, and oxidative stress.

In the TM, multiple miRNAs (e.g., miR-29b and miR-200a) exert anti-fibrotic effects by targeting pathways like TGF-β and Wnt/β-catenin to counteract ECM accumulation (Keller and Peters, 2022; Luna et al., 2009; Luna et al., 2011; Luna et al., 2012; Yu et al., 2022). miRNAs such as miR-143/145 and miR-200c regulate TM cell contractility and IOP, with knockout studies demonstrating reduced IOP and improved outflow in mice (Luna et al., 2012; Li et al., 2017). Others, including miR-18a-5p, also target TGF-β2 signaling to reduce contractility and fibrosis (Knox et al., 2022). Beyond IOP regulation, miRNAs like miR-182, miR-100, and miR-96 influence RGC survival and offer neuroprotection (Kong et al., 2014; Li et al., 2019; Wang and Li, 2014), while others such as miR-27a modulate oxidative stress and inflammatory responses (Tabak et al., 2021).

miRNAs in ocular fluids show promise as biomarkers. Profiles in aqueous humor and plasma are disease-specific, aiding in subtype differentiation (Drewry et al., 2018; Hindle et al., 2019; Rao et al., 2020; Raga-Cervera et al., 2021; Tanaka et al., 2014; Kosior-Jarecka et al., 2021). For example, specific miRNAs like miR-125b-5p are differentially expressed in POAG versus PXG (Drewry et al., 2018). A plasma panel of miR-637, miR-1306-5p, and miR-3159 identified glaucoma patients with an AUC of 0.91 (Hindle et al., 2019), and serum miR-210-3p is elevated in POAG (Liu et al., 2019; Zhao et al., 2023). In addition, tear-based miRNAs have shown promise as a non-invasive method for glaucoma screening (Raga-Cervera et al., 2021). Moreover, miRNAs identified in aqueous humor (e.g., miR-143-3p, miR-125b-5p, and miR-1260b) have been proposed to serve as drug targets (Martinez and Peplow, 2022; Zhang et al., 2023). The functional roles and diagnostic potential of key identified miRNAs are summarized in Table 2.

**TABLE 2:** Functional roles and differential expression of specific miRNAs in glaucoma.

Functional role	miRNAs involved	Sample/Source	Postulated mechanism/Relevance	References
Anti-Fibrotic/ECM Regulation in TM	miR-26a, miR-29b, miR-139, miR-155, miR-200a/c, miR-18a-5p	Human TM cells, animal models	Downregulate ECM components; inhibit TGF-β/Wnt signaling; reduce outflow resistance	Luna et al. (2009), Luna et al. (2011), Luna et al. (2012), Yu et al. (2022), Knox et al. (2022)
TM Contractility and IOP Regulation	miR-143/145, miR-200c	Human TM cells, mouse KO models	Regulate actin cytoskeleton; KO reduces IOP, increases outflow facility	Luna et al. (2012), Li et al. (2017)
RGC Survival and Neuroprotection	miR-182, miR-100, miR-96	RGC-5 cells	Modulate apoptosis, oxidative stress; promote survival via AKT/ERK pathways	Kong et al. (2014), Li et al. (2019), Wang and Li (2014)
Oxidative Stress and Inflammation	miR-27a, miR-182	Retina, tears	Modulate inflammatory/oxidative responses; potential tear biomarker	Tabak et al. (2021)
Differential expression of fluid-based circulating miRNAs	miR-125b-5p	Aqueous humor	Down in POAG, up in PXG	Drewry et al. (2018)
	miR-637, miR-1306-5p, miR-3159	Plasma	AUC = 0.91 for glaucoma detection	Hindle et al. (2019)
	miR-210-3p	Serum/plasma	Elevated in POAG	Liu et al. (2019), Zhao et al. (2023)
	miR-26b, miR-152, miR-30e, miR-151a	Tear	AUC > 0.75 to differentiate between POAG and OHT	Raga-Cervera et al. (2021)

Abbreviations: TM: trabecular meshwork; RGC: retinal ganglion cell; ECM: extracellular matrix; IOP: intraocular pressure; POAG: Primary Open-Angle Glaucoma; PXG: pseudoexfoliation glaucoma; OHT: ocular hypertension; KO: knockout; AUC: area under the curve.

#### 2.6.2 LncRNAs

LncRNAs are transcripts over 200 nucleotides that often act as miRNA sponges (Mattick et al., 2023). By impairing the biological activity of miRNAs, lncRNAs increase the production of proteins from the target mRNAs and their dysregulation is implicated in glaucoma. Key lncRNAs like *MALAT1*, *ANRIL* (also known as *CDKN2B-AS1*), and others (ENST00000552367 and NR_038379) influence TM cell proliferation, ECM remodeling, and RGC vulnerability (Burdon et al., 2018; Zhang et al., 2021; Huang et al., 2022; Pasquale et al., 2013). Xie et al. (2019) suggested that lncRNAs T267384, ENST00000607393, and T342877 might be potential therapy biomarkers for POAG. Some, including NR003923, H19, and LINC00028 are prospective targets for preventing post-surgical fibrosis (Yu et al., 2022; Zhu et al., 2020; Zhao et al., 2019; Sui et al., 2020). However, the functions and associated mechanisms of the role of most lncRNAs in glaucoma are not completely clear yet.

#### 2.6.3 CircRNAs

Similarly, circRNAs are closed-loop molecules that sequester miRNAs to regulate gene expression (Wawrzyniak et al., 2018). A recent study demonstrated that the circRNA *ZRANB1* is expressed in glial cells and it negatively regulates miR-217, promoting Müller cell proliferation and RGC apoptosis (Wang et al., 2018). The expression of c*ZRANB1* was upregulated in glaucoma-induced retinal degeneration, and its knockdown provided a protective effect by reducing retinal gliosis and RGC apoptosis. This effect was reversed by overexpression of RUNX2 (Wang et al., 2018). Targeting this c*ZRANB1*/miR-217/RUNX2 network has neuroprotective potential (Wang et al., 2018). Recent research has identified some candidate circRNAs in ocular hypertension glaucoma models by high-throughput sequencing (Chen et al., 2020); however, the role of most circRNAs in glaucoma remains to be elucidated (Choudhari et al., 2025).

## 3 Translational and clinical potential

**FIGURE 2:**
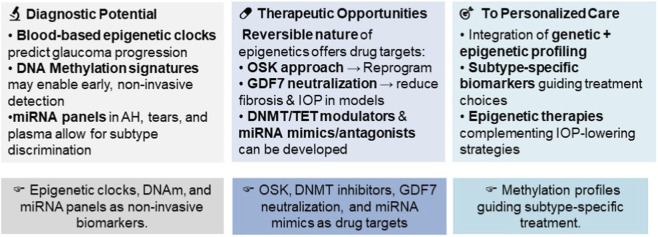
Translational and clinical potential of epigenetics in glaucoma. Schematic representation of the pathways through which epigenetic research is transitioning from mechanistic insight to clinical application, highlighting opportunities in diagnostics, therapeutics, and personalized care.

The epigenetic mechanisms discussed in this review have significant translational and clinical potential to improve glaucoma diagnosis, prognosis, and treatment (Figure 2). Epigenetic aging derived from blood samples (EpiScore) could serve as non-invasive biomarkers of disease susceptibility (Medeiros et al., 2025). Likewise, DNA methylation profiles (e.g., hypermethylation of *LOXL1* in PXG or *CDKN2B* in NTG) and circulating miRNA panels in aqueous humor, tears, or plasma may enable early detection and differentiation between glaucoma subtypes (Greene et al., 2020; Burdon et al., 2018; Martinez and Peplow, 2022). The inherent reversible nature of epigenetic modifications makes them attractive drug targets for clinical intervention. For instance, the epigenetic reprogramming of RGCs using the OSK approach could be the future paradigm for glaucoma treatment to promote RGC survival and restore vision (Lu et al., 2020; Karg et al., 2023). Similarly, with the demonstrated proof-of-concept, GDF7 neutralization might emerge as a promising anti-fibrotic therapy in glaucoma (Wan et al., 2021). In addition, the development of key epigenetic enzyme inhibitors (e.g., DNMT or HDAC inhibitors) and RNA-based therapies (miRNA antagonists) may precisely rectify dysregulated glaucoma pathways in the TM and retina, to reduce fibrosis, lower IOP, and protect the RGCs.

## 4 Challenges and future perspectives

Despite the exciting avenues of epigenetics, there exist several challenges to effectively translate the potential of epigenetics to patient care. The current evidence is mostly based on preclinical and small-scale human studies, highlighting the need to validate the diagnostic and prognostic potential of biomarkers like EpiScore, DNA methylations, and miRNA panels in a diverse population-based longitudinal cohorts. To address these challenges, future research must utilize longitudinal studies and Mendelian randomization to ascertain whether these epigenetic marks are a primary drivers of glaucoma or are secondary consequences of the disease process and cellular stress. Integrating multi-omics data (genomics, epigenomics, and transcriptomics) will be essential to validate these markers as causative agents of disease progression. Furthermore, the high degree of tissue-specific origins of epigenetic changes also presents a significant problem. Consequently, mechanistic studies are required to determine if these changes in accessible samples, such as blood or tears, accurately reflect localized changes in the TM or the optic nerve.

Development of advanced ocular drug delivery systems, such as nanoparticle-based eye drops or viral vectors for CRISPR-based epigenetic editing, is highly crucial for safe and effective application of epigenetic modulators (e.g., OSK factors, *GDF7*, DNMT/HDAC inhibitors, miRNA mimics). Additionally, studies to investigate the effect of environmental factors like diet, exercise, and lifestyle on the ocular epigenetics would be vital steps towards preventative medicine. The ultimate goal will be to incorporate validated genetic and epigenetic data with routine clinical markers, such as IOP, visual fields, and optical coherence tomography, to pave the way for personalized medicine in glaucoma.

## 5 Conclusion

In conclusion, epigenetics redefines our understanding of glaucoma from a static pressure disorder to a modifiable disease at the interface of genetics and environment. Although there are challenges in terms of validation, causality, and drug delivery, the reversible nature of epigenetic mechanisms provides a powerful therapeutic opportunity. Future studies are needed to address these limitations to utilize epigenetics as a source of novel biomarkers, specific drug targets, and personalized care strategies to prevent vision loss beyond IOP control alone.
